# Supplementary figures and images for: Cost-effectiveness of Transcendental Meditation (TM) for treating Post-Traumatic Stress Disorder (PTSD)
Source: PLoS One. 2025 Feb 6;20(2):e0316995. doi: 10.1371/journal.pone.0316995 (PMC11801526; doi:10.1371/journal.pone.0316995)

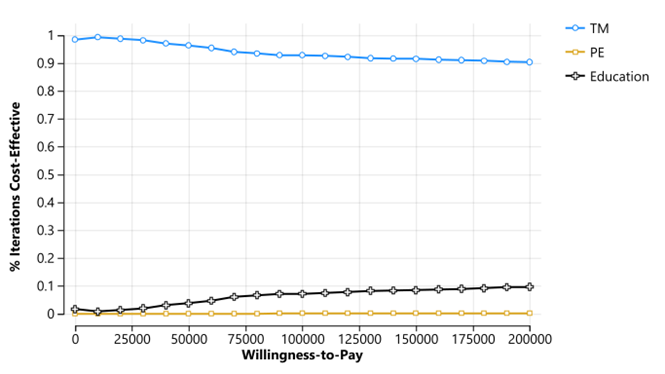

Supplement: S1 Fig — (BMP) [file pone.0316995.s002.bmp]
